# The Impact of Poststroke Aphasia on Quality of Life: A Comparative Cross-Sectional Study

**DOI:** 10.7759/cureus.66988

**Published:** 2024-08-16

**Authors:** Anupama Kurup, Andrea Alby, Anna M Saju, Anuja Anil, Anuja Jayan, Aparna Chandrababu, Arfaz Nazer, Ravi Sankaran

**Affiliations:** 1 Department of Physical Medicine and Rehabilitation, Amrita School of Medicine, Kochi, IND

**Keywords:** hbot, hyperbaric oxygen therapy, quality of life, aphasia, stroke

## Abstract

Objectives: The study aimed to analyze the impact of aphasia on quality of life (QoL) in persons with ischemic stroke per radiological severity, compare equally severe but nonaphasic stroke survivors, and analyze the impact of hyperbaric oxygen therapy (HBOT) exposure*.*

Methods: Patients with first-ever middle cerebral artery (MCA) stroke were categorized by radiological severity into high, intermediate, and low Alberta Stroke Program Early CT Score (ASPECTS). The Stroke Aphasia Quality of Life (SAQoL) Scale was used for outcome analysis. Inclusion criteria were age 40-65, 12-16 months after stroke, MCA distribution, first stroke, and ischemic stroke. Exclusion criteria were mixed vessel involvement and concomitant neurological, orthopedic, or psychiatric comorbidities.

Results: Among 93 patients with ischemic stroke, 87% presented with intermediate-to-low ASPECTS. According to the SAQoL, locomotion and transfers were the most compromised. QoL was significantly negatively correlated with higher ASPECTS and greater stroke impact in those with aphasia overall (p = 0.001). Those who received HBOT overall were significantly better than those who did not, regardless of group (p = 0.02 and 0.03).

Conclusion: The present study shows that the radiological severity of stroke relates to QoL in those with poststroke aphasia. Among those with equal radiological severity, those with aphasia are worse off. Those who receive HBOT have better QoL.

## Introduction

Aphasia is a communication disorder associated with impairments in spoken language, understanding, reading, and writing. It impacts daily activities, participation in society, and the quality of life (QoL) of those with the condition and their family members. Poststroke aphasia can be a crippling deficit in survivors. We performed this comparative cross-sectional study to gauge its impact on QoL by comparing it to equally affected survivors without aphasia. There were 6.5 million stroke cases in India, accounting for 7.1% of total deaths in 2016 [1]. Approximately 30%-35% of stroke victims have aphasia acutely [2]. Of that, in 60%, it becomes chronic [3]. The impact of aphasia on QoL remains inadequately studied from the perspective of radiological severity. Persons with aphasia (PWAs) may have a limited verbal vocabulary for the rest of their lives, hence the relevance of this study. Studies on hyperbaric oxygen therapy (HBOT) in stroke suggest a benefit. To date, no studies have mentioned the impact of HBOT on aphasia. However, they report better QoL and functional recovery [4].

The primary objective of this study is to analyze the overall differences in QoL for those with aphasia compared to those without specifically considering radiological severity. The secondary aim is to examine the impact of aphasia in QoL per Alberta Stroke Program Early CT Score (ASPECTS) subgroups and what tasks were most impaired. Finally, we considered the overall impact of HBOT on QoL.

## Materials and methods

We conducted a cross-sectional observational study from January to December 2021. After obtaining permission from the ethics committee, the hospital stroke registry was reviewed for patients with first-ever middle cerebral artery (MCA) strokes. Inclusion criteria were age 40-65, 12-16 months after stroke, MCA distribution, first stroke, ischemic stroke, and aphasia at presentation. Exclusion criteria were MCA with other vessel involvement and concomitant neurological, orthopedic, or psychiatric comorbidities. All PWAs received speech therapy during acute inpatient rehabilitation. After obtaining patient consent, trained interviewers delivered the Stroke Aphasia Quality of Life (SAQoL) Malayalam version [5]. When the patient could not phonate or indicate an answer on the 5-point Likert scale, the family assisted. All subjects provided informed consent. The following demographic data were collected: age, gender, ASPECTS, handedness, and comorbidities. Patients were divided by the side of the stroke into left and right MCA distribution (as a comparator). They were further subdivided by severity (rated by ASPECTS) into high (8-10), intermediate (4-7), and low (0-3). QoL data retrieved from the database were grouped accordingly and submitted for statistical analysis. Patients were again stratified by whether they received HBOT, and analysis was done for them separately.

HBOT was delivered with 100% O_2_ at a 2-atmosphere absolute pressure in a multiplace chamber, with two 30-minute sessions and a five-minute air break, not including compression and decompression timing. Treatment was started when the patient arrived in the medical ward and was otherwise stable. Recipients underwent 10 dives, along with standard deficit-oriented rehabilitation measures.

Chi-square was used for categorical variables, and the mean with standard deviation was used to represent the outcome variables (Statistical Package for the Social Sciences software, version 21.0, IBM Corp., Armonk, NY, and R software, version 3.3.1, R Core Team, Vienna, Austria). Absolute frequencies and percentages were determined for categorical variables, while the means and standard deviation were established for numerical variables. SAQoL values were compared using the Mann-Whitney test to compare variables with two categories.

## Results

In total, 93 individuals consented. Among them, 78% were males. Of these, 49.6% had left MCA distribution. There were no differences between the groups at baseline. Demographics are detailed in Table 1. With respect to the high and low ASPECTS subgroups, overall, SAQoL was better in the right MCA group but not significant. The intermediate ASPECTS group's overall SAQoL was significantly better than that of the right MCA group (p = 0.04). The domains that showed the most differences were the communication score and psychosocial score (p = 0.02 and 0.03). Each subscore was significantly different, favoring the right MCA group (Table 2). The most affected domain was the physical. This was common between both groups and persisted in the subgroup analysis regardless of groups and subgroups. The most difficult tasks were getting dressed, taking a bath, taking the stairs, and working around the house. There was no significant difference between the groups for this. The least affected domain overall was psychosocial, but this differed between the groups (left and right MCA) and persisted in the intermediate ASPECTS subgroup (p = 0.02).

**Table 1:** Demographic details Data have been represented as mean ± SD and raw numbers. The level of significance of alpha was 0.05 for all analyses undertaken (p = 0.05). LMCA: left middle cerebral artery; RMCA: right middle cerebral artery; ASPECTS: Alberta Stroke Program Early CT Score; DM II: diabetes mellitus type 2; DLP: dyslipoproteinemia; HTN: hypertension; HBOT: hyperbaric oxygen therapy; SD: standard deviation

Details	LMCA (n = 46, 49%)	RMCA (n = 47, 51%)	p value
Age	56.31 ± 0.3	54.73 ± 1.6	0.72
Gender, M:F	35:11 (76%:24%)	32:15 (68%:32%)	0.56
ASPECTS overall average score	5.64 ± 2.11	6.11 ± 1.78	0.66
High ASPECTS	6 (14%)	7 (14%)	0.81
Intermediate ASPECTS	25 (54%)	23 (48%)	0.88
Low ASPECTS	15 (32%)	17 (36%)	0.79
Comorbidities	DM II-32, HTN-36, and DLP-35 (69%, 78%, and 76%)	DM II-33, HTN-34, and DLP-37 (70%, 72%, and 79%)	0.73
Handedness, L:R	2:44 (<1%:99%)	3:44 (<1%:99%)	0.56
HBOT	15 (32%)	15 (32%)	0.89

**Table 2:** SAQoL total and domain scores by group Data have been represented as mean ± SD and raw numbers. The level of significance of alpha was 0.05 for all analyses undertaken (p = 0.05) ASPECTS: Alberta Stroke Program Early CT Score; LMCA: left middle cerebral artery; RMCA: right middle cerebral artery; SAQoL: Stroke Aphasia Quality of Life; PS: physical score; CS: communication score; PsyS: psychosocial score; SD: standard deviation

ASPECTS subgroup	LMCA (49%)	RMCA (51%)	p value
SAQoL total	175	184	0.75
PS	75 ± 4.33	78 ± 5.45	0.78
CS	32 ± 1.98	34 ± 2.44	0.88
PsyS	68 ± 3.36	72 ± 2.76	0.65
Intermediate			
SAQoL total	89	140	0.04
PS	43 ± 8.12	48 ± 7.78	0.57
CS	18 ± 6.23	33 ± 4.97	0.02
PsyS	28 ± 1.45	59 ± 3.91	0.03
Low			
SAQoL total	56	67	0.56
PS	25 ± 4.44	28 ± 7.23	0.64
CS	10 ± 8.67	14 ± 4.42	0.61
PsyS	21 ± 1.76	25 ± 6.53	0.75

When comparing those who received HBOT, those with high or low ASPECTS did not receive this. This was based on their condition, not warranting more than basic care. All recipients fell into the intermediate ASPECTS category. Regardless of the side, there was a significant difference favoring those who received it (p values: L = 0.04 and R = 0.03). The domain that showed the most difference was psychosocial (p values: L = 0.01 and R = 0.02) (Table 3).

**Table 3:** Intermediate ASPECTS subgroup analysis HBOT: hyperbaric oxygen therapy; SAQoL: Stroke Aphasia Quality of Life; PS: physical score; CS: communication score; PsyS: psychosocial score; ASPECTS: Alberta Stroke Program Early CT Score

Side		HBOT (8)	No HBOT (17)	p value
Left	SAQoL total	132 ± 1.67	93 ± 2.31	0.04
	PS	58 ± 6.23	49 ± 8.22	0.06
	CS	24 ± 3.91	14 ± 4.52	0.05
	PsyS	50 ± 2.43	30 ± 1.25	0.01
Side		HBOT (9)	No HBOT (14)	p value
Right	SAQoL total	166 ± 5.54	115 ± 4.42	0.03
	PS	67 ± 3.27	62 ± 6.12	0.23
	CS	30 ± 1.48	21 ± 7.23	0.06
	PsyS	69 ± 6.34	32 ± 3.78	0.02

## Discussion

The results of this study show that those with a left MCA stroke will have worse QoL if the stroke is intermediate to low in ASPECTS when compared to peers with similar right MCA involvement. The impact of aphasia was most evident in the intermediate ASPECTS group. The domains showing the most differences were communication and psychosocial. The results also reveal that HBOT will lead to better QoL overall. The high ASPECTS group, though having aphasia at admission, made a nearly full recovery, which is expected considering radiological severity. This is why their communication scores, while lower than the right-side group, were not significant. The low ASPECTS group had poorer functional levels overall, resulting in aphasia being the least of their issues. The intermediate group, on the other hand, had significant enough recovery for aphasia to be problematic.

Aphasia is a communication disorder associated with impairments in spoken language, understanding, reading, and writing that impact daily activities, participation in society, and the QoL of those with the condition and their family members. The cost of treating aphasia is approximately €6905.87 [6]. Compared with no therapy, its outcomes are improved functional communication, reading, writing, and expressive language [7]. Before this, the impact of aphasia on QoL was not reported using a radiological severity score or a comparator group. Using the ASPECTS allows a clinician to plan rehabilitation. The comparator group exposes the impact of aphasia. Past studies on aphasia and QoL do not generally use scales designed to accommodate aphasia. When they do, they do not have the available language comparability for our population. We intended to isolate communication as a domain and see if it persisted as a problem. Using this scale also helped sort out the effects of HBOT. The results show that impaired communication also affects the psychosocial domain.

It has been reported that as many as 50%-70% of stroke survivors regain functional independence. Still, many are left with permanent disabilities due to the isolated or combined effects of hemiparesis (50%) that may affect walking (30%) and the use of upper extremities for activities of daily living (ADLs) (26%), depression (35%), and communication/aphasia (19%) [8]. The results of our study confirm with these findings. Existing studies are underpowered or do not have a control group to exhort the impact of aphasia. Moller reported QoL using the World Health Organization Quality of Life Brief version on eight individuals [9]. Rangamani and Judovsky conducted a study on 21 PWAs that showed QoL scales do not address communication-related QoL [10]. SAQoL is validated and covers the aspects we want to consider. A study on the impact of chronic aphasia in persons with stroke reported difficulty with ADLs and dissatisfaction overall [11]. It was a cross-sectional semistructured interview using the profile of functional activities and life participation as a proxy for the functional independence measurement/functional activity measurement and International Classification of Functioning. They compared 40 affected individuals in two different communities. Another study was the accounts of four female stroke survivors with aphasia. It showed highly personal details from the participants' lives [12]. Spaccavento et al. had a comparator group of PWAs, but their study was used to validate a QoL questionnaire [13].

The International Classification of Function was used to frame this issue with semistructured interviews of 20 families having stroke survivors with aphasia [14]. It showed that families with affected persons restructure grocery shopping and vacations. It generally impacts coping and naturally affects communication and methods of conveying basic common issues. Family members had less time for their health and well-being, increased domestic work, and less time for social/community activities. A systematic review [15] supported the same.

Having a stroke impacts QoL, even if it is mild [16]. A different study showed that the side of stroke had little impact on health-related quality of life (HRQOL) outcomes. It also showed women were worse off six months after the event [17]. The studies mentioned have patients with mild strokes, meaning aphasia was not an issue.

Self-care, mobility, upper extremity function, work, and productivity are the domains where the survivors show significant decline [18]. The results of our study support these findings. Even when the dominant hand was preserved, transfer and locomotion issues persisted.

Multidisciplinary physiatrist-led rehabilitation has a strong positive effect on QoL [2]. These gains may be lost six months after discharge, meaning the skills gained do not translate into daily life [17]. This likely factored into our results also. Adapting poststroke is more than just physical capacity. Transition back to one's pre-event environment may reinforce the sense of lost capacity. The subsequent low-functioning state leads to a sense of less well-being. This can be augmented by social support and educational resources [18,19]. Of those, the most significant predictors of HRQOL included marital status, quality of social support, functional status, and depression [20].

India has a conspicuous absence of physiatrists, and paramedicals capable of treating aphasia are also in deficit. Larger centers may be capable of offering acute care, but maintenance care is a challenge. Regardless of the treatment of aphasia, earlier is better. A systematic review of telerehabilitation for aphasia shows it is on par with face-to-face care [21]. Exploratory studies employing artificial intelligence for aphasia have also begun. It can help triage patients' needs, facilitating care provision [22].

No prior studies have looked at the impact of HBOT and aphasia. The results show that the communication scores are significantly better in the groups receiving it, regardless of the side. This is not unexpected, considering that studies show the overall benefits of this intervention in stroke [4]. A meta-analysis reports that Orgogozo Scale and Trouillas Disability scores were better with HBOT [23]. If HBOT made a difference, then why is one side better than the other? HBOT recipients were only of the intermediate ASPECTS subgroup. The eloquent cortex cannot be regrown. Neural plasticity recruits penumbra neurons, and angiogenesis from HBOT facilitates this. Gains were mostly in psychosocial and communication. The difference was not in the motor domain. While the HBOT left MCA group did significantly better, this remained the most affected domain. How did this modality affect the change? There is a high density of blood vessels/cm^2^ of brain tissue [24]. In stroke, acute oxygen consumption is increased [25]. HBOT induces angiogenesis and reduces inflammatory mediators like hypoxia-inducible factor‑1 alpha, matrix metallopeptidase 9, cyclooxygenase-2, Nogo-A, and myeloperoxidase [26]. This results in the repair and recovery of cellular damage.

## Conclusions

Those with a left MCA stroke will have worse QoL if the stroke is intermediate to low in ASPECTS when compared to peers with similar right MCA involvement. The impact is most noted in the intermediate ASPECTS group. This group had significant enough recovery for aphasia to be problematic. The QoL domains showing the most differences were communication and psychosocial. No prior studies have looked at the impact of HBOT and aphasia. The results show that the communication scores are significantly better in the groups receiving it regardless of the side. It did not affect the motor domain. While the HBOT L MCA group did significantly better, this remained the most affected domain.
